# Internal conversations, self-reliance and social support in emerging adults transitioning from out-of-home care: An interpretative phenomenological study

**DOI:** 10.1177/13591045211005827

**Published:** 2021-04-22

**Authors:** Peter Appleton, Isabelle Hung, Caroline Barratt

**Affiliations:** 1 School of Health and Social Care, University of Essex, Colchester, UK; 2 Pain Management Centre, National Hospital for Neurology and Neurosurgery, University College London Hospitals, UK

**Keywords:** Self-reliance, reflexivity, care leavers, social support, social relationships, internal conversations, emerging adulthood, Interpretative Phenomenological Analysis

## Abstract

Young people transitioning from out-of-home care frequently have a history of maltreatment and multiple psychosocial challenges. ‘Survivalist self-reliance’ – thought to involve social disconnection from others, and reluctance to seek support – provides one coping strategy. However, little is known about the self-reliant young person’s own reflexive interpretations of social relationships and support during transition. This qualitative study addresses the question: In the context of transitioning from out-of-home care, what reflexive meanings do ‘avowedly’ self-reliant individuals attribute to current social support and social relationships? Participants were four avowedly self-reliant young adults in transition from care, each with a history of maltreatment and multiple adversities. In this secondary analysis, data were from semi-structured interviews utilizing Margaret Archer’s internal conversations interview framework. Data were analyzed using Interpretative Phenomenological Analysis (IPA). Three thematic contexts were identified in which social support was salient: (a) current thoughts and active memories of both the birth family and foster families; (b) the importance of socializing; and (c) perceptions of formal services. There was evidence of cognitive reappraisal (a known amenable resilience factor) and selective engagement with social support, despite the strong overall stance of self-reliance. The findings suggest a more nuanced approach to our understanding of ‘survivalist self-reliance’.

## Introduction

Adolescents and young adults transitioning from out-of-home care are known to experience multiple psychosocial challenges. Previous history of maltreatment, removal from home, placement changes and educational disruptions may be compounded by the challenges of transition itself (Akister et al., 2010; Hiles et al., 2014). Young people in transition from foster care are known to be two to four times more likely to experience lifetime and/or past year mental health disorders, compared to transition-age young people in the general population (Havlicek et al., 2013).

Transition services (including ‘extended care’) for care-leavers have gradually emerged in some jurisdictions, but there is a need for more interdisciplinary and international research into psychosocial need in order to generate evidence-based practice (van Breda et al., 2020). A recent international review of transition services policy notes: ‘The overwhelming majority of youth in the transition from care to adulthood in the countries in our sample are left to survive on their own at age 18 or younger, even when legislation makes provisions for them to stay in care longer’ (Strahl et al., 2021). It is perhaps not surprising that a policy of ‘self-reliance’ has been found to be important for young people themselves in these circumstances.

In a paper from the Midwest study of young people ageing out of foster care (Samuels & Pryce, 2008) ‘survivalist self-reliance’ or ‘vigilant self-reliance’ characterized the identities of many young people in their study. This pattern of self-reliance was described as being composed of: early developmental conferral of adult-like independence; maltreatment; gradual recognition by the young person that others do not share this experience; a gradual social disconnection of young people from important others; and, during transition, reluctance to seek support. In a later study, Pryce et al. (2017) found that the process of help-seeking was a ‘central struggle’ for self-reliant young people leaving care, reflecting a number of relational processes, beginning with the emotional impact of early family breakdowns, continuing with frequent placement moves, and compounded by relational disconnection from transition caseworkers.

In the UK, Stein (2006) described a sub-group of care-leavers he called ‘survivors’, with histories of significant instability, frequent placement moves and further disruption during transition. These young people saw themselves as self-reliant (Stein, 2006).

Although we do not know the prevalence of high self-reliance among care-leavers (Samuels and Pryce’s paper implies a high prevalence; Stein suggests an important sub-group), these studies have identified a form of experience among some care-leavers which may have profound implications for the young people’s own futures, and has implications for the design of accessible and evidence-based services.

For those providing or planning services for care-leavers, a question arises: How do self-reliant individuals, in transition from care, appraise current social support and social relationships – both informal and formal? We know that high self-reliance implies a *general* tendency to avoid help-seeking and perhaps to avoid the development of social relationships, but what might we discover if we take a ‘very close look’ at experiential aspects of (cognitive) re-appraisal and re-evaluation of social support and social relationships (Best & Blakeslee, 2020; Wade, 2008), specifically among those who appear especially self-reliant, in the context of transition from care?

## Internal conversations, reflexivity, social support and survivalist self-reliance

Our starting point is the work of sociologist Margaret Archer, who has developed a theoretical framework focusing on the agential role of reflexivity in social functioning (Archer, 2003, 2007). For her:

At its most basic, reflexivity rests on the fact that all normal people talk to themselves within their own heads, usually silently and usually from an early age. In the present book this mental activity is called ‘internal conversation’ but is also known *inter alia* as ‘self-talk’ - - -

(Archer, 2007, p. 2)

People’s reflexive sense of what matters – what is subjectively important to them as individuals – makes it possible to plan, participate and function in society (Archer, 2003). It follows that the internal conversations of young people who regard themselves as particularly self-reliant would be of interest (and practical relevance) in the context of severe and sometimes repeated ruptures between young people and their formative social and emotional support systems (Barratt, Appleton & Pearson, 2020; Hung & Appleton, 2016). In a qualitative study of internal conversations, using Archer’s semi-structured internal conversations interview (Archer, 2003, pp. 161–162), in a heterogeneous sample of care-leavers (age 19–23) in London, UK, four individuals out of the sample (total *n* = 9) were markedly and avowedly self-reliant (Hung & Appleton, 2016). They described themselves, during the internal conversations interview, as highly independent in their own internal conversations and way of life. In each case, the interviews were infused with (a) a sense of independent thinking *and* (b) detailed accounts of the reflexive links participants made between their independent world-view and their ‘owned’ adverse histories (which included, for instance, specific accounts of abuse, neglect, further abuse in care, estrangement from family, issues with services).

In the primary study, we described these individuals’ avowal of high self-reliance, and their rich autobiographical ‘justifications’ for this stance, but we did not examine experiential aspects of (cognitive) re-appraisal and re-evaluation of current social support and social relationships. The present study, involving a secondary analysis of data on these four avowedly self-reliant individuals, extends the original study in two ways: (a) using Interpretative Phenomenological Analysis (IPA), we focus on the phenomenology and hermeneutics of the self-reliant young people’s reflexive descriptions of current social support and social relationships, (b) we extend our theoretical perspective, initially based on the work of Margaret Archer, to include other perspectives.

Thus, the current study addresses the question: In the context of transitioning from out-of-home care, what reflexive meanings do ‘avowedly’ self-reliant individuals attribute to current social support and social relationships?

## Method

### Participants

Nine participants took part in the original study, of whom four defined themselves as self-reliant and survival-oriented (Charelle (female, age 20), Danny (male, age 20), Don (male, age 23), Nailah (female, age 21): Hung & Appleton, 2016). The names are pseudonyms. This new secondary analysis focuses on these four individuals. They had left care between 6 months and 6 years previously. The participants were diverse in terms of ethnicity, sexual orientation, number of placements, current accommodation, education, and involvement with the criminal justice system. No participants were currently accessing mental health services – however, three out of the four discussed in detail recent or current significant psychiatric symptoms. All four self-reliant participants discussed their memories of maltreatment and/or neglect.

### Ethics

The study had ethical approval from the University of Essex, and the UK Social Research Ethics Committee. Further details are available in the original study paper (Hung & Appleton, 2016).

### Interviews

The semi-structured qualitative internal conversations interview followed the guidelines described by Archer (2003, pp. 161–162). The interview has two sections, which we administered in the form of two separate interviews (for full details of the interview see Hung & Appleton, 2016).

The purpose of the first section – our first interview – is to enable the young person to discuss their experience of internal conversations. The interviewer emphasizes that ‘we are all different’, and ‘there are no right answers’ – many people experience conversations with themselves, or ‘self-talk’, silently in their heads. I’d like to know whether this is so for you and if you could tell me a bit about this experience? Following any individual examples of internal conversations offered by the participant the interviewer provides ten prompts: planning, rehearsing, mulling over, deciding, re-living, prioritizing, imagining, clarifying, imaginary conversations, budgeting (see Archer, 2003, p. 161, for definitions of each prompt).

In the second section (our second interview) the researcher asks the question: ‘Which areas of your life matter most to you at the moment’? The question allows the young person to discuss ‘what matters’ or what is important in their life, entirely from their own reflexive perspective, building on internal conversations discussed in the first section. In Archer’s interview protocol there then follows a section which focuses on ‘how each subject internally deliberated about his or her own future’ (Archer, 2003, p. 162; Appleton, 2020; Hung & Appleton, 2016).

### Analysis

Returning to the original transcripts for this secondary analysis, an Interpretative Phenomenological Analysis (IPA) approach was used (Smith et al., 2009). IPA provides an approach that emphasizes people’s active meaning-making (Smith, 2019). Using IPA for analysis had the benefit of a coherent ‘epistemic fit’ with the form of the data, which were gathered in a semi-structured interview context in which young people’s own reflexive interpretations of their social worlds were a primary focus (Archer, 2003).

But meaning-making isn’t just for participants – IPA emphasizes the double hermeneutic – ‘the researcher trying to make sense of the participant trying to make sense’ (Smith et al., 2009, p. 3). In the interests of transparency of interpretative analysis (Smith, 2019; Tuval-Mashiach, 2017), we describe below how the (secondary) analysis evolved from the research question and the analytical stance.

Initial analysis of active meaning-making (in relation to social support and social relationships) by participants led to an emerging focus on the following five aspects of the interviews: (a) what (or who) is important to the individual participant (discussed explicitly at the beginning of Interview 2, but may also be discussed at any point during the interviews [Appleton, 2020; Archer, 2003]); (b) areas in which the participant discussed relatively rich or detailed reflexive deliberations about social relationships or social support; (c) social relational and social support areas in which the participant had had second thoughts – for example, ‘on reflection, now I understand’; (d) social relational and social support areas in which reframing (Khawaja et al., 2008) was used by the participant; (e) social relational and social support areas in which the participant thought ‘x’, despite salient broader considerations (e.g. working happily with a specific professional, despite a general negative view of the support offered by public services).

After re-familiarizing with each individual transcript in its entirety, notes were made about each reflexive discussion of social support and social relationships in each of the above five analytic contexts.

Semantic patterns, across cases, were then identified (Smith et al., 2009, pp. 101–103). During the analysis process it became clear that these across-case, ‘higher-order’, domains of meaning were not ‘themes’ as such, but were better described as thematic contexts (Riggs, 2018).

By thematic contexts we mean broad thematic areas/common areas of deliberation/of *reflexive* interpretation, *within which there was a wide diversity of individual accounts* in which social support and social relationships were highly salient.

The thematic contexts were as follows: (a) current thoughts and active memories of the birth family and foster families; (b) importance of socializing; and (c) perceptions of formal services.

Differences between individuals were carefully noted at each stage of the analysis and remain integral to the presentation of the findings (Smith et al., 2009, pp. 29–32).

Some participant details have been omitted to protect identity.

## Results

### Current thoughts and active memories of the birth family and foster families

A key finding is that across the four avowedly self-reliant participants there was a very wide range of reflexive interpretation of birth family and foster families. We deliberated as to whether to separate data on birth families and foster families, but kept these in the same thematic context (a) because participants tended to cross-reference their discussions (i.e. between birth family and foster families), and (b) to reflect extant literature on care leavers’ perceptions of family, including wider kin (Biehal & Wade, 1996; Gwenzi, 2020; Wade, 2008).

Danny seemed to be estranged from his family, certainly in the terms of his own internal conversations, which were thoughtfully angry. Talking about his birth family:

My family haven’t provided me [with] moral support since even I was a young age. Not even from a young age. So I know for a fact that I can’t rely on my family.

He discussed the ‘side-taking’ that he currently saw in his family, and commented metaphorically: ‘we’re not in a goddam war where we have to choose which side to be on’, and explained: ‘Family are definitely not in the higher prospects of my life’.

For Charelle, answering the question at the beginning of the second interview: *what matters to you most in your life at the moment?*:

At the moment, myself – myself because obviously I’ve got to think how my rent is being paid, how I’m budgeting, I’ve got to think of myself at the moment.

Clarifying her sense of self being of primary importance, Charelle explains:

Well if I don’t have a roof over my head, I can’t do anything. That is obviously my main thing ‘cos I ain’t got no mum, no guardian, or anyone to look after me so if I ain’t looking after me, who is? No-one else ain’t feeding me, no-one else ain’t bathing me so it’s me, I come first.

Her deep anger is evident in these words. However, anger is not all we hear during the interview. She wants to study arts, and remembers her birth mother telling her how she ‘used to get all the prizes’ for art. She felt this ability was ‘in my blood’.

Charelle also had specific memories of one foster carer, who ‘taught [me] a lot’, and with whom she was in regular ongoing contact.

Nailah felt very distant from her birth family and, like Danny and Charelle, expressed this with thoughtful anger. But she had found a way of ‘reframing’ her expectations, and had some significant reflexive understanding of the intergenerational impact of her parents’ own experience of abuse.

As part of the extended discussion in the second interview about what was important to her, Nailah was asked: *you’ve mentioned your friends and going out – what about your family – where do they come in the list of important things to you?* Nailah replied:

Nothing. They will always be there - - - When did I stop hating them? When I was 19 - - - they’re not important, I just view them as a friend and that’s it - - - you can’t be disappointed if you view them as a friend. Soon as you start viewing them as a parent all they’re going to do is disappoint and upset you.

She discussed her memories of the original abuse she had undergone as a child, but also discussed her own realization (via discussions with other family members) that they – both her parents – had themselves been abused by their parents. She said that ‘it helped me understand’.

She discussed specific and detailed memories of enjoying being with her father, earlier in her life.

Like Charelle she had good memories of one foster carer: one active memory ‘the one that always keep coming up from before’

was one of my last foster carers and the conversations we had. I look back on it and see she was trying to help me and prepare me for things and help me avoid the situation that I’m in right now but I didn’t listen. I was just like “Ah, you’re too much in my business, go away”. I thought I was a big woman, that’s the problem - - - yeah that keeps popping up

Don, different from each of the three other participants, was in touch with most of his extended family, including his birth mother. He reflected in detail on his closeness to some of them, and how there was still ‘building’, ‘re-building’ and extending (i.e. meeting family members he had previously been unaware of) to do. He discussed the importance he gave to these processes via a conversation he remembered having had with his grandmother recently:

Like my Grandmother said last time I saw her: “we don’t let down family, we don’t put down on our family, we have to come together and this needs to be done – give it time – and then it needs to be done”. So I’m trying to give space - - -

In summary, for these individuals who all self-identify as especially self-reliant, there is a very wide spectrum of detailed reflexive interpretation of family and foster families, including, for some individuals, specific positive memories, reframing and/or current active involvement in relationship-building or maintenance. Three out of the four individuals expressed significant anger as they discussed family.

### Importance of Socializing

At the beginning of Interview 2, Nailah included socializing (in addition to higher/further education, and part-time work), in her account of what was currently important to her:

And go out really and release my stress and socialise as that’s the thing I like doing the most (laughs)

And in the first interview, Nailah:

I’m - - - a people’s person - - - I love hearing people’s points of views on stuff.

Danny, similarly, says:

I have lots of people to talk to - - - I like to listen to understand where he’s coming from - - I learn a lot from everyone else, no matter what they speak.

For Danny, going out with friends to a nightclub

makes me feel better about myself as I know that people want me to come and join them.

Gently challenged by the interviewer about how this might suggest that social relationships might be important, Danny ‘saw the point’, and clarified:

I need to be myself, to do the stuff I want to do.

Don included socializing with a named friend, and ‘the rest of my friends’ (as well as family) in his list of most important aspects of his current life.

In summary: socializing and mixing with others was important to three out of the four avowedly self-reliant individuals.

### Perceptions of formal services

Charelle, talking about some of the services she had received:

They can’t help, and to be honest, they put me in most of the bad situations I’m in, so I don’t like asking them. For my housing benefit they messed up. They bribe - - - the way they see it is like we do something for you, so what are you doing for us? - - - they’re the only people I can’t control my anger - - - they play mind games.

She also described the arrangements for seeing her birth mother, who lived in a different part of the country. She explained that services were ‘funny about giving me the money [for travel to see her mother]’ and ‘I kind of blame - - - services in a way because I think they could do more for us young people’. Also thinking of her birth mother, she says: ‘how could you [i.e. services] let her end up homeless when she’s our mum?’ She also felt that services had not helped her stay in touch with her siblings.

However, despite not trusting services in general, Charelle identified three professionals who she trusted fully, knowing what to speak with each about.

Nailah remembered the period running up to leaving care, when she felt that the ‘adult world would be horribly scary’, and that staff ‘put pressure on you to say what you wanna do’. Looking back, she says she recognizes why: ‘cos their manager is putting pressure on them’.

Very recently the paperwork for an application she (Nailah) put in for a specific year-long course, for which she was highly motivated, and remembers having put a lot of preparatory work in, was lost, and the course filled up. She recalls:

They were just like, “Oh sorry we’ll put you on the waiting list and you can apply next year”. That’s not the point!

This administrative error, in the context of her own reflexive awareness of her lengthy history of educational disruptions and difficulties, formed a severe blow to her self-esteem. She described the mental health issues that arose from this episode, making sense of it via her careful assessment of not having much time (i.e. in months and years) to gain educational qualifications in order to obtain work.

However, there is one current teacher/educator she feels close to, whose help she fully recognizes.

In summary: two participants discussed detailed and thoughtfully emotional accounts of how services had been unhelpful, but also identified individual workers whom they trusted and could work closely with.

## Discussion

‘Survivalist’ self-reliance may be understandable as a coping strategy for dealing with a social world that may have included maltreatment, removal from home and further ruptures in relationships and developmental opportunities (Samuels & Pryce, 2008). Furthermore, autobiographical accounts by participants themselves, justifying their strongly ‘owned’ policies, add weight to our understanding of ‘where the young person is coming from’ (Hung & Appleton, 2016).

For those providing help during transition from out-of-home care, this strategy / policy may seem like a ‘closed book’. It seems to cut off potential opportunities for social support. Our research question, focusing on what reflexive sense avowedly self-reliant individuals make of current social support and social relationships, seems, prima facie, to ‘go against the grain’ of the declared policies of the young people. However, the IPA-based secondary analysis brought to light ‘exceptions to the rule’, flexibilities in thinking and feeling, and selective engagement with social relationships. These congregated in three thematic contexts: (a) current thoughts and active memories of the birth family and foster families; (b) importance of socializing; and (c) perceptions of formal services.

In the first thematic context, there was a very wide range of individual differences. Positive memories (D’Argembeau et al., 2003), positive thoughts (Macleod & Moore, 2000), and/or reframing (Khawaja et al., 2008) were evident in the deliberations of three individuals. Charelle and Nailah had positive memories of foster mothers – Charelle was still in regular contact with her foster mother. Charelle had a specific positive memory of her birth mother noticing and valuing her ability in arts – this memory motivated her current educational and vocational intentions. Nailah remembered enjoyable time spent with her father when she was little and had reframed her expectations of family relationships. Don quoted his grandmother giving a positive account of how important family was, a stance he clearly identified with.

The *fun* of socializing was important to two of the participants. Reflexively, Danny identified aspects of personal identity which seemed to be validated by having fun in a group; Nailah identified management of stress.

Another aspect of socializing that came to light in the narratives of two participants was the experience of ‘listening to other people’s points of view’. This finding chimes with ‘post-traumatic growth’ data from Greenberg et al. (2018) suggesting that history of trauma may increase a person’s ability to take the perspective of another.

Two participants discussed detailed and emotionally expressive accounts of how services had been unhelpful, but also identified current individual workers who they trusted and could work closely with. These accounts could not be characterized as ‘pot-shots’ at an agency but were deeply felt reflections on the services that had supposedly had a duty of parental care toward them. However, individual staff were identified whom they could trust. These constituted reflexive exceptions to the general rule of being personally critical of public services.

### Anger

Reflexive thoughts about family, and about formal services, were expressed with considerable emotion, including anger.

The reflexive detail provided by participants suggested a cognitively and affectively complex process of trying to make sense of the existential meaning of family violations of trust, and of public service struggles to meet need. Congdon’s (2018) recent work on resentment and anger as a creative resource is helpful. He offers a formulation of resentment as a ‘transformation, reworking the resenter’s very conception of what is valuable or important in the first place’ (Congdon, 2018, p. 741). We do not have space to do justice to Congdon’s carefully argued paper, except to note that anger may, in some contexts, be a strong indicator of active reflexive work, gradually transforming a personal outlook on normative aspects of the social world.

### What sense do we make of the apparent lack of individual ‘internal consistency’ in high self-reliance?

As indicated above, Stein (2006), in a British study of care-leavers, described a sub-group he called ‘survivors’, who were characterized as highly self-reliant. As part of his formulation of this sub-group, he found that survivors’ ‘view of themselves as independent was often contradicted by the reality of high degrees of agency dependence for assistance with accommodation, money and personal assistance’. This finding, of apparent ‘internal inconsistency’, is borne out in the findings discussed in the present paper.

Indeed, in our study, apparent ‘inconsistency’ (i.e. high self-reliance *and* cognitive reappraisal of, and engagement with, aspects of social support and social relationships) applied not only to the stance toward services (by two participants), but also perceptions of family, and of socializing. How might we formulate this finding?

The philosopher Bratman (2013) has argued that individual human rational agency is usually characterised by norms of consistency – including consistency between particular intentions and an agent’s overall beliefs. In a separate paper on care-leavers’ approaches to rational planning norms (Appleton, 2019) it has been suggested that developmental subjection to violation of norms, including removal from home, and more profoundly the complex violation of relational and physical safety that occurs when maltreatment is present (Warmingham et al., 2019, p. 30), may have led to a healthy implicit scepticism about a range of psychosocial norms. If the young person’s approach to making sense of social support is reflective and thoughtful, and sometimes angry, but not consistent, then perhaps we should regard it as ecologically valid (Morton, 2011), reflecting ‘work in progress’ (Congdon, 2018), making sense of a social world that has routinely flouted norms in its dealings with the young person. Our study throws some preliminary light on the reflexive process of understanding, re-building, and sometimes enjoying social relationships, despite an overall stance of self-reliance.

The data reported here may also be regarded as an example of cognitive reappraisal, a known ‘amenable resilience factor’ that may moderate *and* mediate the relationship between developmental adversity and young people’s mental health status (Fritz et al., 2018).

### Strengths and limitations

One of the strengths, and indeed purposes, of the internal conversations interview is that the participant is invited to reflect on their own internal conversations and identify matters of importance in their own lives (Archer, 2003). IPA, focusing on the analysis of interview transcripts in terms of participants’ (and researchers’) active search for meaning (Smith, 2019), ‘matched’ the reflexive form of the internal conversations data.

The sample size requires justification (Vasileiou et al., 2018). First, IPA is usually conducted with small, purposive and homogenous samples, in which idiographic aspects of data can be teased out (Smith et al., 2009). This is the approach we used here, but with an additional focus on individual reflexive meaning-making, as described by Jonathan Smith in his recent ‘extended theoretical positioning’ on IPA (Smith, 2019); second, the idiographic and micro-textural analysis (discovering, as it turned out, major individual differences between participants) facilitated transparency as regards our own reflexive deliberations during the analysis (Tuval-Mashiach, 2017); third, the original data-set provided a special opportunity to study experiential meaning-making of apparently highly self-reliant participants about social relationships during the transition from care – the sample size was dictated by the design of the original study, but, perhaps more importantly, this sample was what we have termed ‘avowedly’ self-reliant – self-reliance seemed to be part of their ‘declared’ personal identity (Hung & Appleton, 2016).

The question of generalizability arises with very small samples. While we hope that some of our discussion about the role of apparent ‘inconsistency’ and anger may be of ‘general’ value in advancing understanding of self-reliance during the transition from care (Vasileiou et al., 2018), we are also committed to the expectation and hope that different demographic and cultural contexts are likely to be found to be associated with different forms of self-reliance and different personal stances toward social support among young people in transition from care (van Breda & Pinkerton, 2020).

### Implications for practice, and collaborative assessment of amenable resilience factors

In this small-scale study, points for practice would include the importance of offering avowedly self-reliant young people the opportunity to talk in more specific detail about their thoughts and feelings about current experience of actual and potential social support (informal and formal) and social relationships in order to tap potentially rich reflexive discussions (and cognitive reappraisal) about social context (Best & Blakeslee, 2020; Fritz et al., 2018; Ungar, 2013), while also acknowledging understandable parallel anger about a range of forms of maltreatment and service failures to meet need (Congdon, 2018; Pryce et al., 2017).

There may be a particular relevance for Local Authorities (who hold a statutory duty of care in the UK context) to continue to develop ways of ensuring the voices of care-leavers are heard by services (Dixon et al., 2019).
